# Identification of a N6-Methyladenosine (m6A)-Related lncRNA Signature for Predicting the Prognosis and Immune Landscape of Lung Squamous Cell Carcinoma

**DOI:** 10.3389/fonc.2021.763027

**Published:** 2021-11-18

**Authors:** Chengyin Weng, Lina Wang, Guolong Liu, Mingmei Guan, Lin Lu

**Affiliations:** Department of Medical Oncology, Guangzhou First People’s Hospital, School of Medicine, South China University of Technology, Guangzhou, China

**Keywords:** m6A (N6-methyladenosine), long noncoding RNA, lung squamous cell carcinoma (LSCC), prognosis, immune microenvironment

## Abstract

**Background:**

m6A-related lncRNAs emerged as potential targets for tumor diagnosis and treatment. This study aimed to identify m6A-regulated lncRNAs in lung squamous cell carcinoma (LUSC) patients.

**Materials and Methods:**

RNA sequencing and the clinical data of LUSC patients were downloaded from The Cancer Genome Atlas (TCGA) database. The m6A-related lncRNAs were identified by using Pearson correlation assay. Univariate and multivariate Cox regression analyses were utilized to construct a risk model. The performance of the risk model was validated using Kaplan–Meier survival analysis and receiver operating characteristics (ROC). Immune estimation of LUSC was downloaded from TIMER, and the correlations between the risk score and various immune cells infiltration were analyzed using various methods. Differences in immune functions and expression of immune checkpoint inhibitors and m6A regulators between high-risk and low-risk groups were further explored. Finally, Gene Ontology (GO) and Kyoto Encyclopedia of Genes and Genomes (KEGG) analyses were utilized to explore the biological functions of AL122125.1.

**Results:**

A total of 351 m6A-related lncRNAs were obtained from TCGA. Seven lncRNAs demonstrated prognostic values. A further multivariate Cox regression assay constructed a risk model consisting of two lncRNAs (AL122125.1 and HORMAD2-AS1). The Kaplan–Meier analysis and area under the curve indicated that this risk model could be used to predict the prognosis of LUSC patients. The m6A-related lncRNAs were immune-associated. There were significant correlations between risk score and immune cell infiltration, immune functions, and expression of immune checkpoint inhibitors. Meanwhile, there were significant differences in the expression of m6A regulators between the high- and low-risk groups. Moreover, GO and KEGG analyses revealed that the upregulated expression of AL122125.1 was tumor-related.

**Conclusion:**

In this study, we constructed an m6A-related lncRNA risk model to predict the survival of LUSC patients. This study could provide a novel insight to the prognosis and treatment of LUSC patients.

## Introduction

Lung cancer, as the most prevalent malignancy, ranks as the leading cause of cancer-related deaths worldwide (1, 2). Non-small cell lung cancer (NSCLC), as the major histological type, accounts for 85% of lung cancer. Lung squamous cell carcinoma (LUSC) accounts for 30% NSCLC (3, 4). Currently, the targeted therapy and immunotherapy mainly benefit the non-LUSC NSCLC (5, 6). The 5-year survival rate for LUSC patients remains unsatisfactory (7). Therefore, identifying novel biomarkers for diagnosis and treatment is of great potential.

Long noncoding RNAs (lncRNAs) are non-coding transcripts with a length longer than 200 nucleotides (8, 9). lncRNA regulates gene expressions in transcriptional and post-transcriptional mechanisms (8, 10, 11). Although lncRNAs have been used as biomarkers in predicting survival and treatment response in LUSC (12, 13), more and more research started to investigate lncRNA correlated to cellular functions, such as ferroptosis-related lncRNA (14–16) and autophagy-related lncRNA (17, 18).

N6-Methyladenosine (m6A), as the most abundant and reversible RNA modification, is involved in the regulation of RNA splicing, localization, stability, and translation (19, 20). m6A modification could regulate the expression and biological processes of mRNAs and non-coding RNAs (21, 22). The m6A-regulated process was involved in three kinds of m6A regulators, which include methyltransferases (writers), demethylases (erasers), and m6A-binding proteins (readers). Gu et al. identified three m6A regulators (WTAP, YTHDC1, and YTHDF1) as independent prognostic factors for LUSC patients (23). Liu et al. found that FTO was correlated with poor overall survival of LUSC patients (23). m6A-related lncRNAs demonstrated promising roles in tumor diagnosis, prognosis, and treatment. Yu et al. constructed an m6A-related lncRNA signature which could accurately predict the survival of kidney renal clear cell carcinoma patients (24). Moreover, m6A regulators also demonstrated potential therapeutical roles in disease treatment (25), such as FTO inhibitors MO-I-500 (26) and FB23-2 (27). However, whether m6A-related lncRNAs are involved in LUSC progression needed to be elucidated.

In the present study, we identified m6A-related lncRNAs with independent prognostic value to construct a risk model. The correlations of the risk model with the immune microenvironment were further studied.

## Materials and Methods

### Data Source and Retrieve

Transcriptome profiling data and corresponding clinical data were downloaded from The Cancer Genome Atlas (TCGA) database (https://portal.gdc.cancer.gov). A list of 23 m6A regulators was obtained from a published article, which include writers (METTL3, METTL14, METTL16, WTAP, VIRMA, RBM15, RBM15B, and ZC3H13), erasers (FTO and ALKBH5), and readers (YTHDC1, YTHDC2, IGF2BP1, IGF2BP2, IGF2BP3, YTHDF1, YTHDF2, YTHDF3, HNRNPC, LRPPRC, HNRNPA2B1, FMR1, and RBMX) (24, 28, 29). The differential analysis of m6A regulators and m6A genes was obtained using “limma” package in R software.

### Identification of m6A-Related lncRNAs

Firstly, differentially expressed lncRNAs were retrieved using “limma” package. Then, correlations between these lncRNAs and m6A regulators were analyzed using Pearson correlation analysis. m6A-related lncRNAs were selected with the following criteria: coefficient >0.4, *P <*0.001. The results were demonstrated by a network graph using “igraph” package in R.

### Construction and Validation of an m6A-Related lncRNA Risk Model

Univariate and multivariate Cox regression analyses were performed to screen m6A-related lncRNAs with prognostic values using “survival” package. Ultimately, we identified two prognostic m6A-related lncRNAs to construct a risk model. The risk score was calculated by coefficient(1) * lncRNA(1) expression + coefficient (2) * lncRNA(2) expression. Then, the LUSC patients were classified into high-risk and low-risk groups based on the median risk score. The Kaplan–Meier survival curve was used to evaluate the prognostic capability of the risk model. The area under the curve (AUC) value of the receiver operating characteristics (ROC) was used to assess the sensitivity and specificity of the risk model. The distributions of risk scores, survival status, and these two m6A-related lncRNA were visualized by “pheatmap” package.

### Immune Estimations of the Risk Model

Tumor immune estimation resource was downloaded from TIMER (http://timer.cistrome.org/) (30, 31). The immune infiltration estimations were analyzed by TIMER, CIBERSORT, quanTIseq, xCell, MCP-counter, and EPIC algorithms. The correlations between the risk score and immune functions and the expression of checkpoint inhibitors were demonstrated by a package in R software.

### Correlation Between Risk Model and m6A Regulators

The differential expression of the 23 m6A regulators between the high- and low-risk groups was analyzed using a package in R software.

### Gene Ontology and Kyoto Encyclopedia of Genes and Genomes Analysis

Gene Ontology (GO) enrichment analysis was performed to annotate the functions of AL122125.1, which included biological process (BP), cellular component (CC), and molecular function (MF) in R software. Kyoto Encyclopedia of Genes and Genomes (KEGG) enrichment analysis was performed to reveal the associated signaling pathways. GO and KEGG enrichment analyses were performed by using “colorspace”, “stringi”, “clusterProfiler”, “org.Hs.eg.db”, and “pathview” packages in R software.

### Statistical Analysis

Data processing was performed by Perl programming language. The statistical analysis was carried out in R software (version 4.1.0). *P*-value <0.05 was considered as statistically significant.

## Results

### Expression of m6A Regulators in LUSC

**Figure 1:**
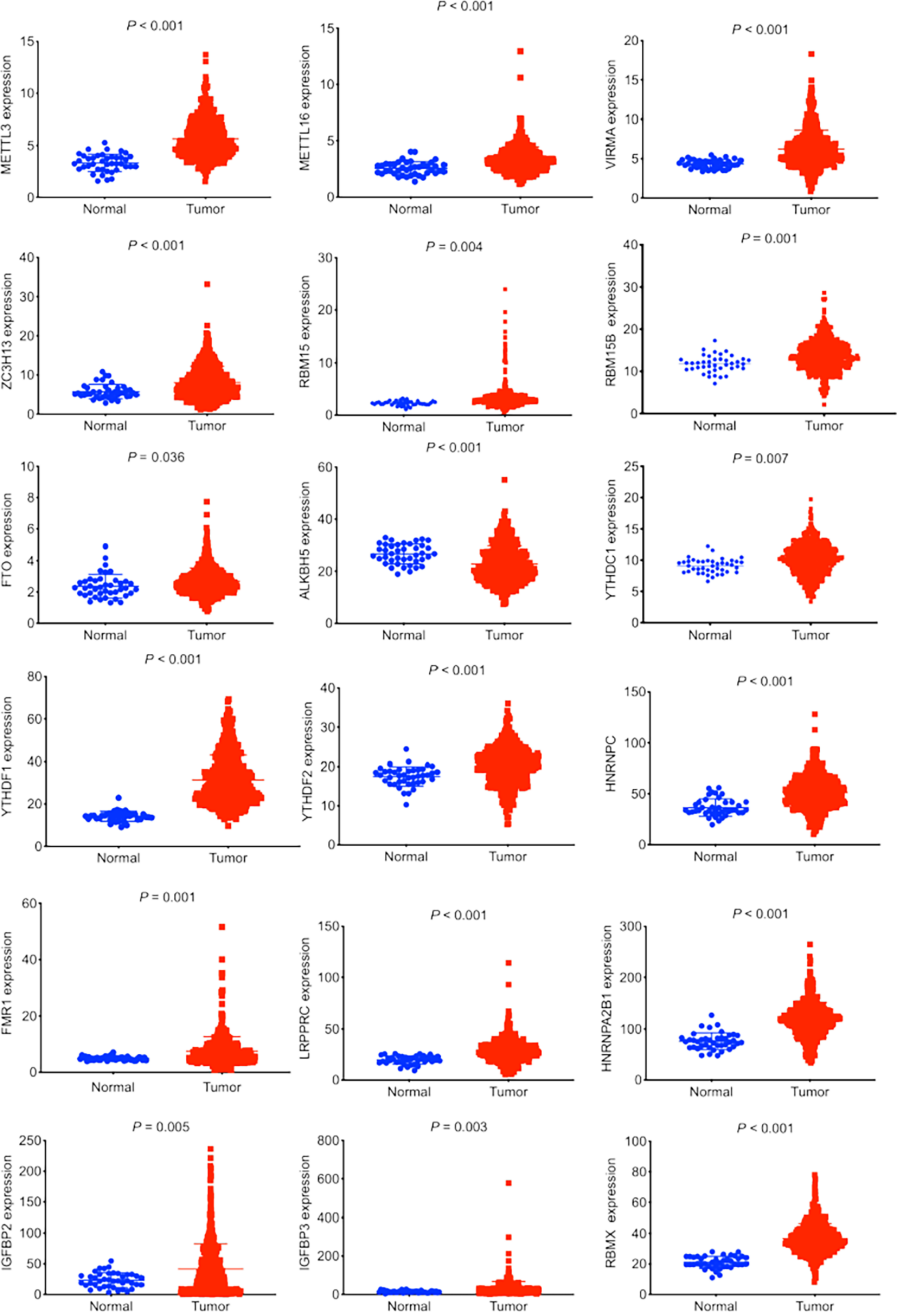
Expression profiles of m6A regulators in lung squamous cell carcinoma (LUSC). The data was retrieved from The Cancer Genome Atlas database. The expression of 23 m6A regulators between LUSC tumor tissues and normal tissues was compared. Eighteen out of 23 m6A regulators demonstrated significant differences in expression.

In total, there were 501 LUSC tumor tissues and 48 normal tissues in this study. According to a literature search, we investigated the expression profiles of 23 m6A regulators in LUSC. An upregulated expression of 17 m6A regulators (METTL3, METTL16, VIRMA, ZC3H13, RBM15, RBM15B, FTO, YTHDC1, YTHDF1, YTHDF2, HNRNPC, FMR1, LRPRPC, IGFBP2, IGFBP3, and RBMX) was observed in LUSC tumor tissues (**Figure 1**). The downregulated expression of m6A regulator ALKBH5 was observed in tumor tissues (**Figure 1**).

### Identification of m6A-Related lncRNAs in LUSC

**Figure 2:**
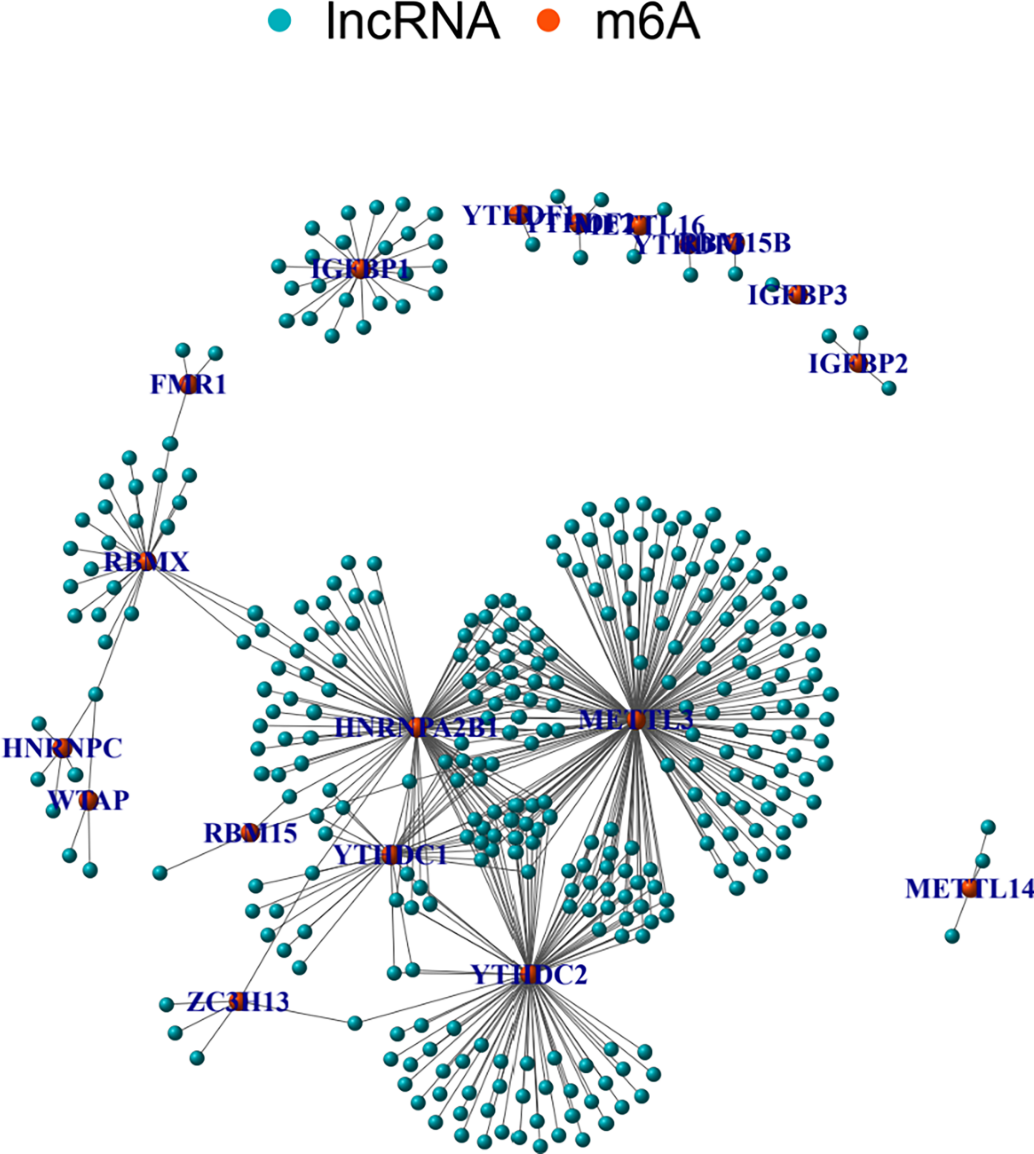
m6A-related lncRNAs. Graphs summarizing the m6A-related lncRNAs.

The correlations between m6A regulators and lncRNAs were calculated using Pearson correlation coefficient. There were 351 lncRNAs related to m6A regulators. **Figure 2** shows a network graph of the lncRNAs correlated to m6A regulators. As shown in **Figure 2**, 18 m6A regulators were significantly related to various lncRNAs. There were 188 lncRNAs demonstrating significant correlations with METTL3. Other m6A regulators demonstrated less correlated lncRNAs.

**Figure 3:**
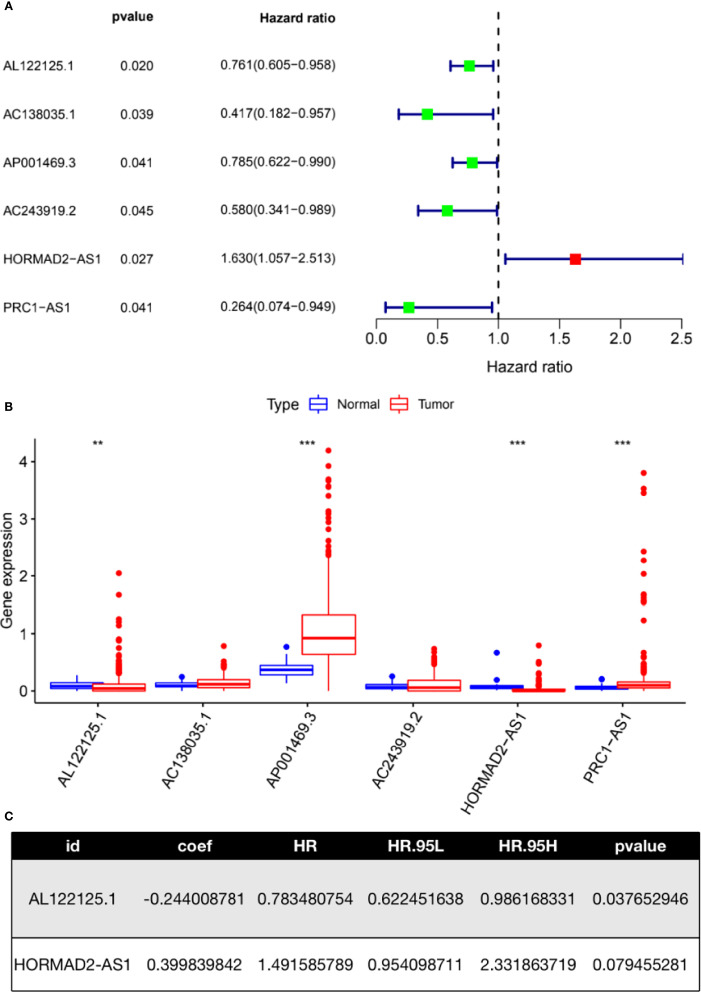
Construction of an m6A-related lncRNA risk model. **(A)** Univariate Cox regression was used to identify m6A-related lncRNAs with prognostic value. **(B)** Expression of m6A-related lncRNAs identified from univariate Cox regression analysis. **(C)** Multivariate Cox regression assay was used to identify m6A-related lncRNAs with an independent prognostic value. **P < 0.01; ***P < 0.001.

Univariate Cox regression analysis was performed to filter lncRNAs with prognostic values. There were six lncRNAs (AL122125.1, AC138035.1, AP001469.3, AC243919.2, HORMAD2-AS1, and PRC1-AS1) in the TCGA-LUSC cohort that were significantly correlated with the survival of LUSC patients (**Figure 3A**). **Figure 3B** shows the differential expression of these lncRNAs. As we can see, there were significant differences in the expression of AL122125.1, AP001469.3, HORMAD2-AS1, and PRC1-AS1 between LUSC tumor tissues and normal tissues. A subsequent multivariate Cox regression analysis selected two m6A-related lncRNAs (AL122125.1 and HORMAD2-AS1) to construct a risk model (**Figure 3C**).

**Figure 4:**
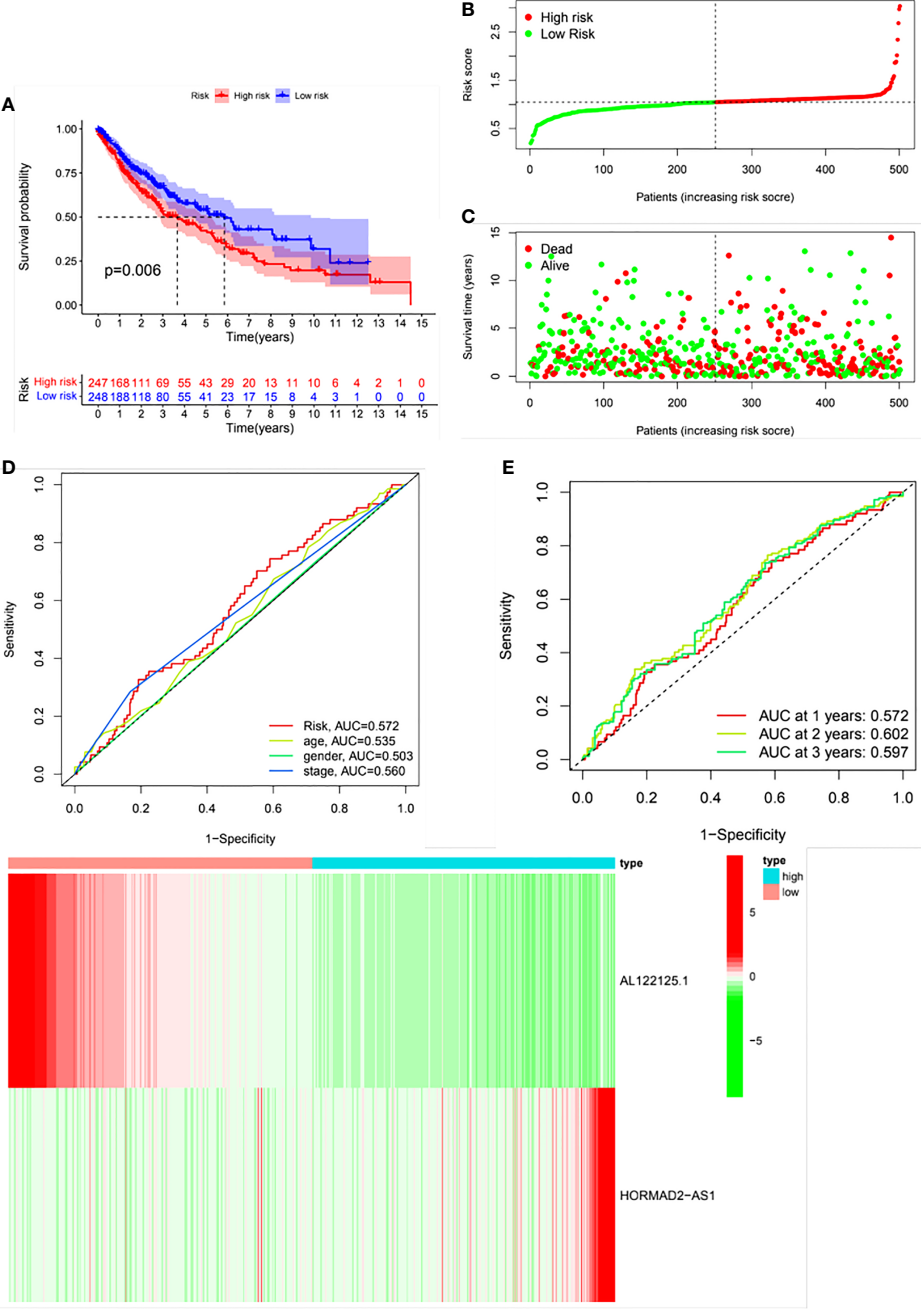
Validation of the risk model. Patients were divided into high- and low-risk groups based on the risk score. **(A)** Kaplan–Meier survival curve of the risk model. **(B)** Distribution of the risk scores. **(C)** Distribution of the survival status. **(D)** Receiver operating curve (ROC) of the risk score and conventional clinical factors. **(E)** ROC curves of the risk score in predicting 1-, 2-, and 3-year survival. **(F)** A heat map of the differential expression of AL122125.1 and HORMAD2-AS1 the between high- and low- risk groups.

LUSC patients were classified into high- and low-risk groups based on the median risk score. The risk score could well predict the survival of LUSC patients. As shown in **Figure 4A**, the Kaplan–Meier survival curve showed that there was a significance difference in the survival of high- and low-risk groups. **Figures 4B, C** depict the distribution of risk scores and survival status in the two groups. The ROC curve was conducted to assess the performance of this risk model in predicting the survival of LUSC patients. As shown in **Figure 4D**, the AUC results indicated that this risk model demonstrated better sensitivity and specificity than conventional clinical risk factors (age, gender, and stage). The AUC values for 1, 2, and 3 years were 0.572, 0.602, and 0.597, respectively (**Figure 4E**). **Figure 4F** shows the relative expression of the two m6A-related lncRNAs between the high- and low- risk groups.

### Correlations Between the Risk Model and Immune Microenvironment

**Figure 5:**
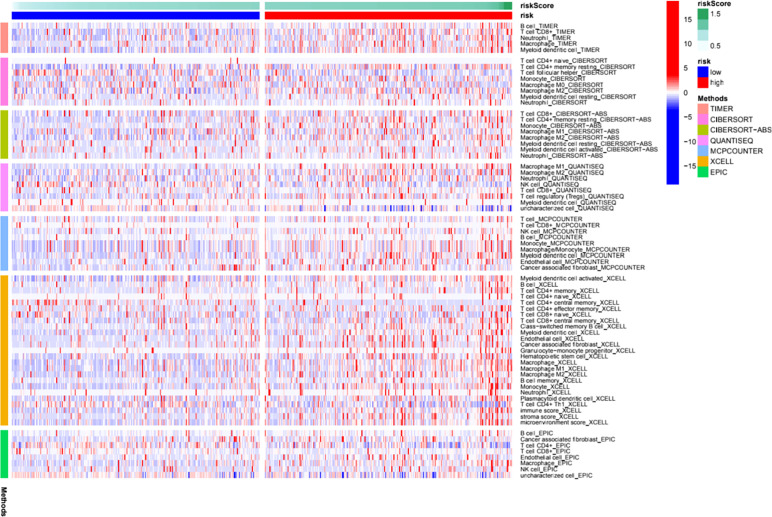
Immune cell infiltration features in the high- and low- risk groups. A heat map of the differences of immune cell infiltrations between the high- and low-risk groups.

**Figure 6:**
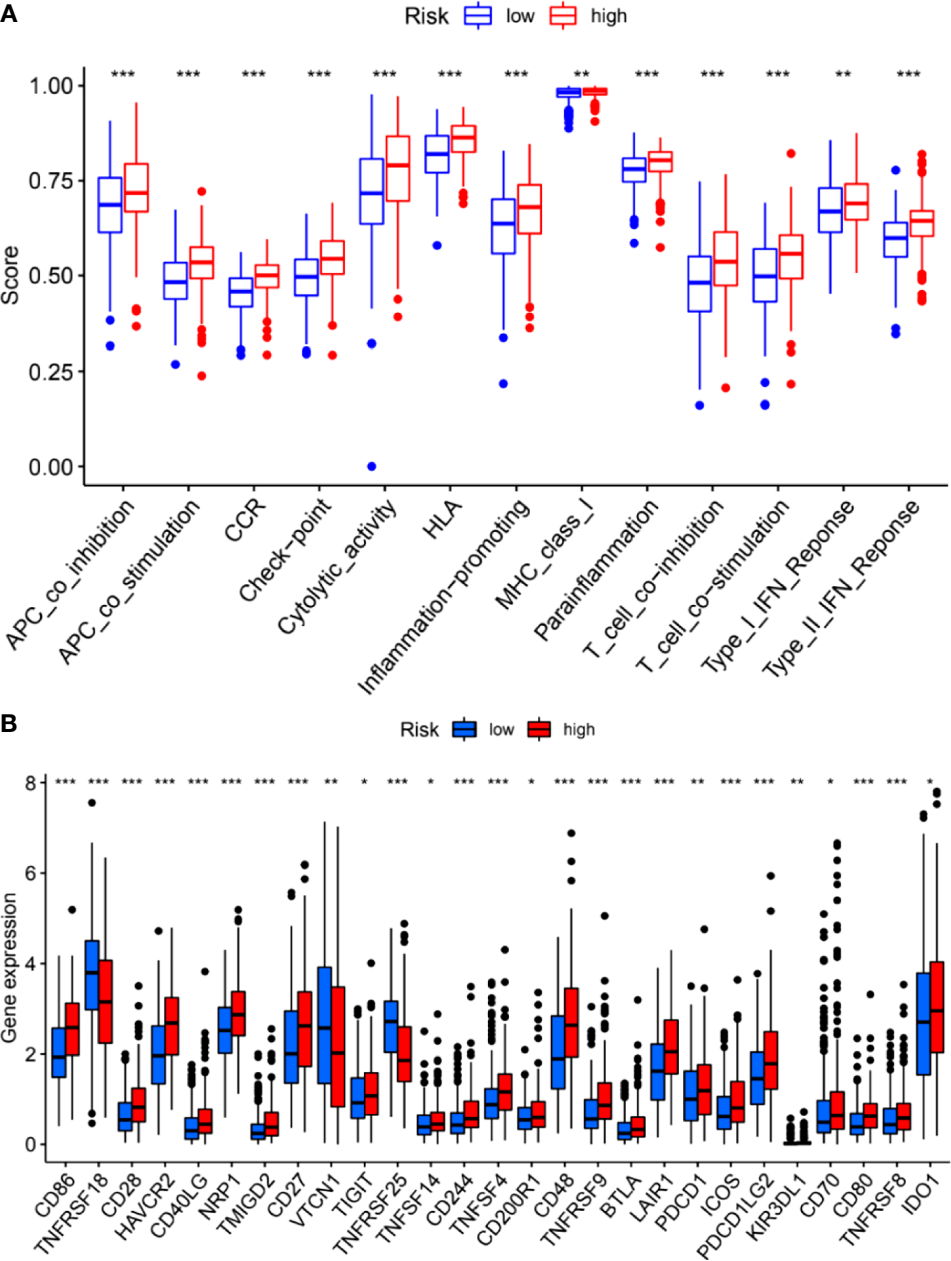
Immune functions **(A)** and expression of immune checkpoint inhibitors **(B)** between the high- and low-risk groups. *P < 0.05; **P < 0.01; ***P < 0.001.

We further investigated the correlation between the m6A-related lncRNA risk model and the immune microenvironment. The immune estimation data of the TCGA cohort was downloaded from TIMER. The infiltration of immune cells demonstrated prominent differences between the high- and low-risk groups. **Figure 5** shows the infiltration of immune cells, which were calculated by TIMER, CIBERSORT, quanTIseq, xCell, MCP-counter, and EPIC algorithms. The immune functions in the high- and low-risk groups were assessed using R software. As shown in **Figure 5**, different algorithms demonstrated different immune cell infiltration between the high- and low-risk groups. Some immune cells demonstrated significant differences according to most of the algorithms, such as B cell, T cell CD8+, neutrophil, macrophage, and NK cell. Moreover, the two groups demonstrated significant differences in APC co-inhibition, APC co-stimulation, CCR, check-point, cytolytic activity, Type I IFN response, *etc.* (**Figure 6A**). Moreover, the differences in the expression of immune checkpoint inhibitors, such as CD86, TIGIT, BTLA, *etc.*, are demonstrated in **Figure 6B**. Thus, these findings indicated that the m6A-related lncRNA model may have potential roles in predicting the immune response.

### Correlations Between the Risk Model and m6A Regulators

**Figure 7:**
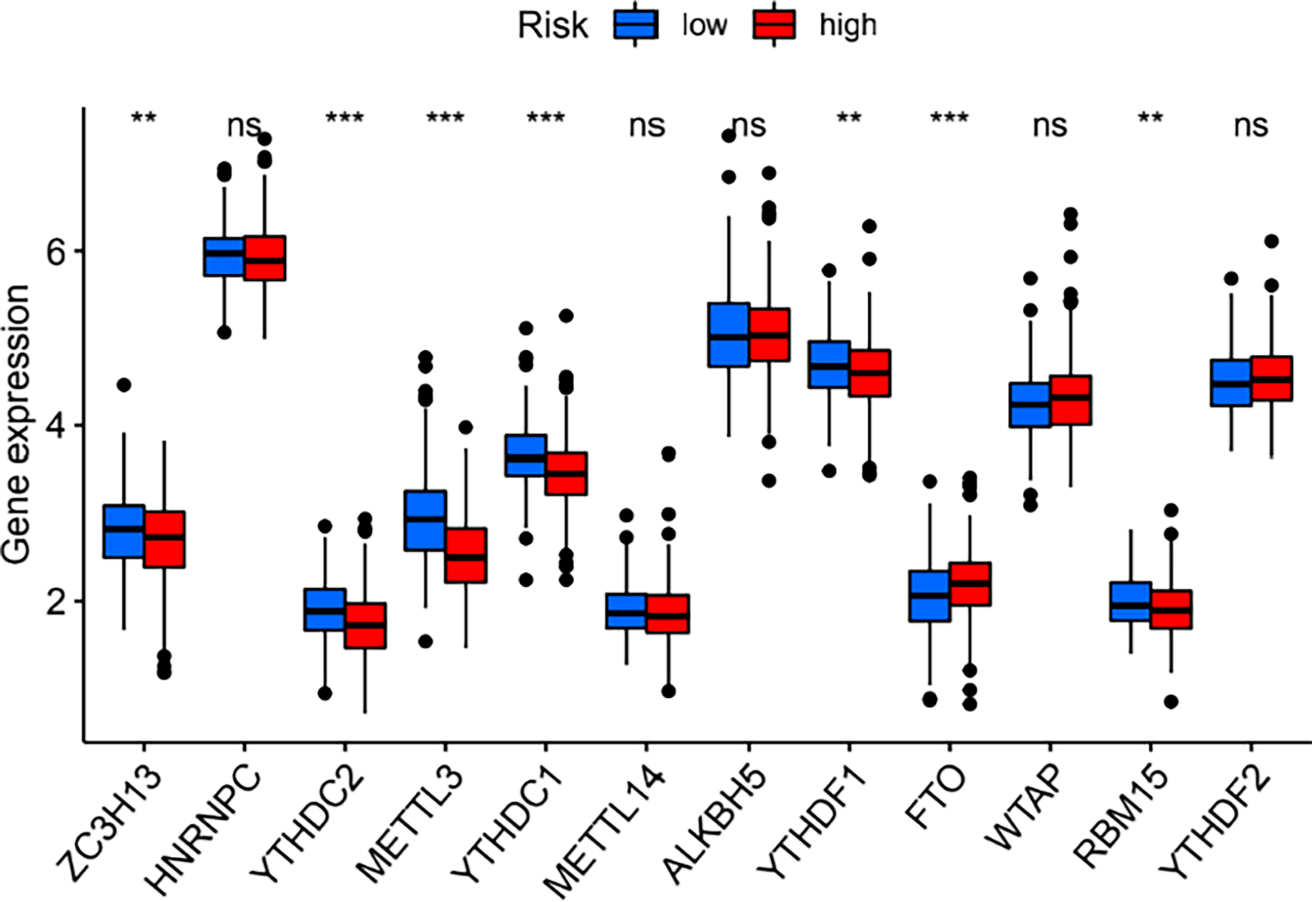
Differences in the expression of m6A regulators between the high- and low-risk groups. **P < 0.01; ***P < 0.001. ns, no significance.

The expression of the 23 m6A regulators between the high- and low-risk groups was compared. As shown in **Figure 7**, the expression of ZC3H13, YTHDC2, METTL3, YTHDC1, YTHDF1, and RBM15 was significantly downregulated in the high-risk group, while FTO expression was significantly upregulated in the high-risk group.

### Expression and Biological Roles of AL122125.1

**Figure 8:**
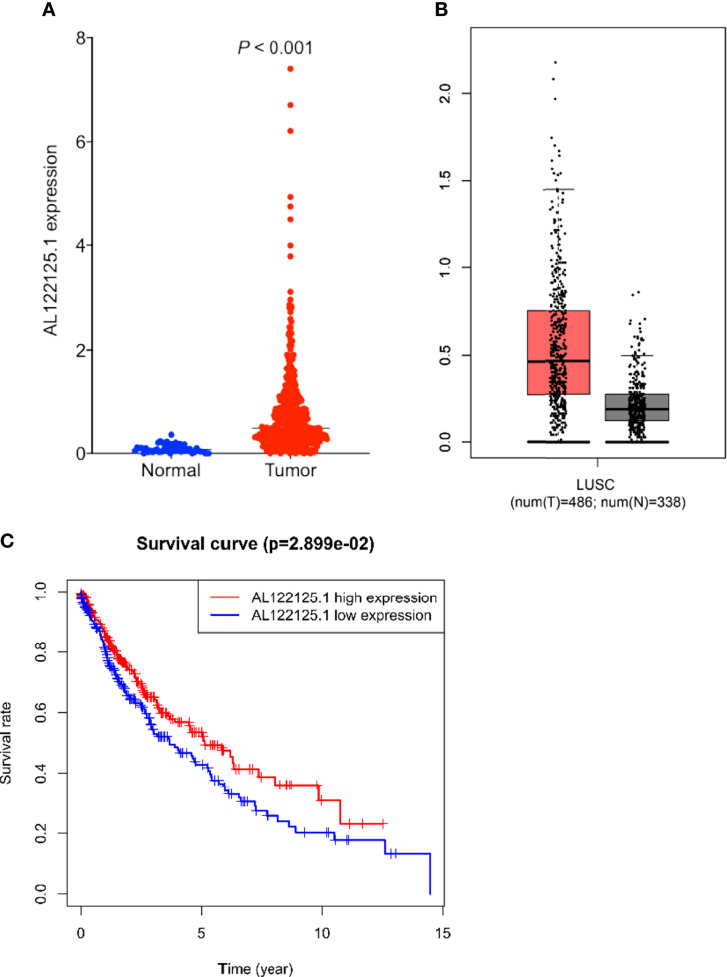
Expression and prognosis value of AL122125.1. The differential expression of AL122125.1 was retrieved from The Cancer Genome Atlas (TCGA) **(A)** and Gene Expression Profiling Interactive Analysis **(B)**. **(C)** Kaplan–Meier survival curve of AL122125.1. Data was retrieved from TCGA database.

Multivariate Cox regression results showed that AL122125.1 was an independent prognostic factor for LUSC patients. We further explored the expression, prognostic value, and biological functions of AL122125.1. Both TCGA and Gene Expression Profiling Interactive Analysis database showed the upregulated expression of AL122125.1 in LUSC tumor tissues (**Figures 8A, B**). Kaplan–Meier survival analysis revealed that the upregulated expression of AL122125.1 was correlated with poor overall survival of LUSC patients (**Figure 8C**).

**Figure 9:**
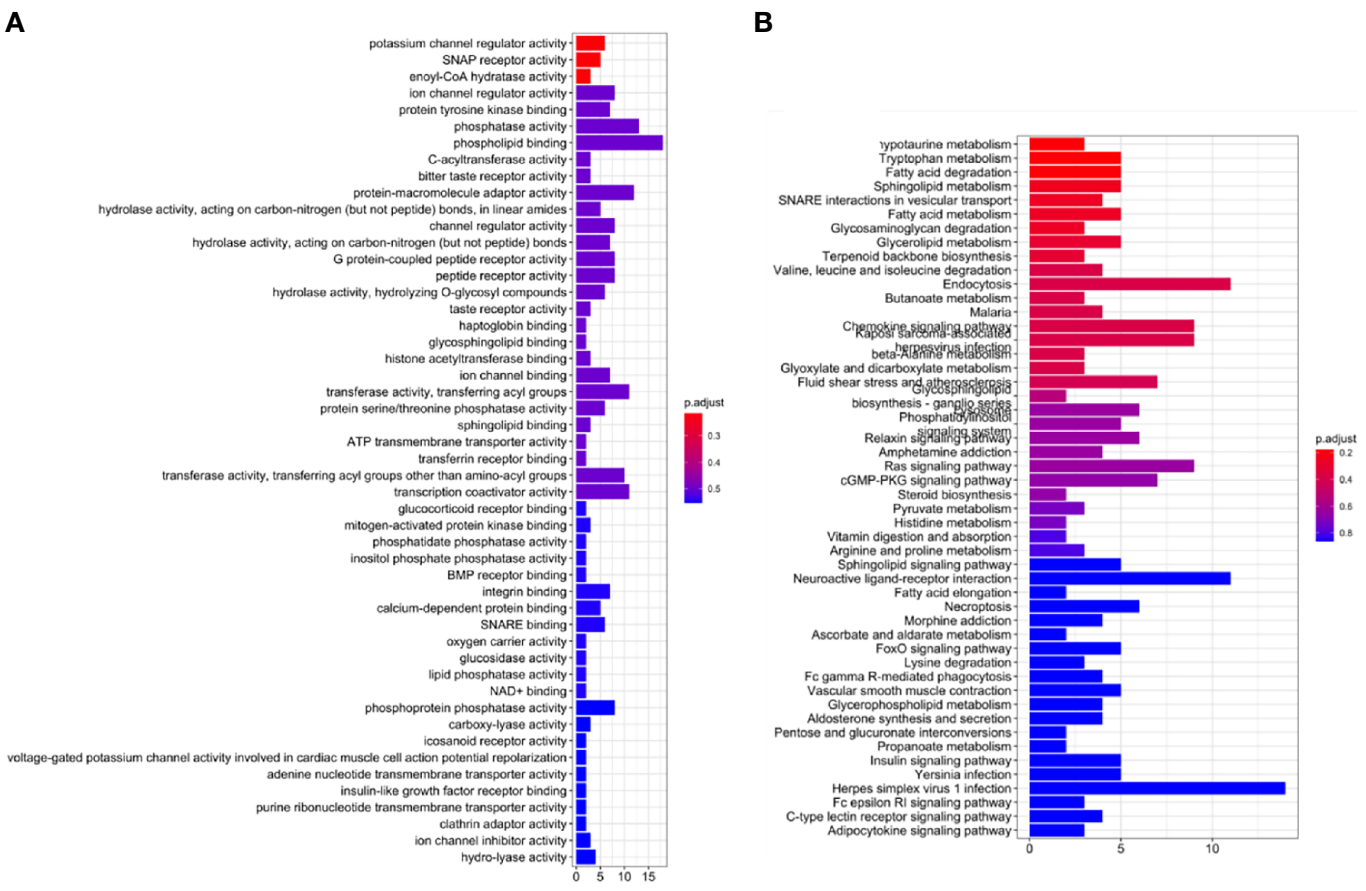
Functional analysis of AL122125.1. **(A)** Gene Ontology enrichment analysis of AL122125.1. **(B)** Kyoto Encyclopedia of Genes and Genomes enrichment analysis of AL122125.1.

To explore the underlying mechanisms of AL122125.1, GO enrichment analysis and KEGG pathway analysis were performed. As shown in **Figure 9A**, the GO results showed that AL122125.1 was related to ion channel regulator activity, protein tyrosine kinase binding, histone acetyltransferase binding, and ATP transmembrane transporter activity. Further KEGG pathway enrichment analysis showed that AL122125.1 was involved in many tumor-related pathways and metabolic pathways (**Figure 9B**).

## Discussion

Unlike lung adenocarcinoma, the treatment strategies for LUSC patients are limited (23). Thus, identification of novel biomarkers could provide novel strategies for LUSC patients. Many biomarkers, such as lncRNAs and m6A genes, have emerged to have important roles in tumor diagnosis and treatment (13, 15). RNA modification plays crucial roles in the transcriptional and post-transcriptional regulation of gene expression (19, 22). m6A modification is the most common RNA modification form (19, 22). Meanwhile, there was a close correlation between lncRNAs and m6A regulators (24, 29). Their interaction could regulate the expression of target genes as well as the cellular biological functions (21, 32, 33). Thus, in this study, we identified m6A-related lncRNAs to construct a risk model in LUSC. The risk model was closely correlated to immune microenvironment.

Firstly, differentially expressed m6A regulators were screened between LUSC tumor tissues and normal tissues. We identified a dysregulated expression of 18 m6A regulators. Increasing studies revealed that m6A modification of lncRNAs could regulate the progression of various tumors, and lncRNAs might regulate the expression of m6A regulators (34). We next identified a total of 351 m6A-related lncRNAs from the TCGA-LUSC cohort. Univariate and multivariate Cox regression analyses were accordingly applied to construct an m6A-related lncRNA risk model. Here we identified two m6A-related lncRNAs, AL122125.1 and HORMAD2-AS1. There were no studies that reported the prognostic values and biological functions of AL122125.1 and HORMAD2-AS1 in tumors. Both m6A-related lncRNAs were revealed for the first time. Thus, there are a lot of work that need to be carried out.

LUSC patients were classified into high- and low-risk groups based on the constructed risk model. The KM analysis revealed that the high-risk group demonstrated poor overall survival than the low-risk group. Although the AUC values for 1-, 2-, and 3-year OS were 0.572, 0.602, and 0.597, respectively, the AUC values for the risk model seemed better than the conventional clinical parameters. These results suggested that this risk model still demonstrated good sensitivity and specificity.

The immune microenvironment includes various immune cells and secreted factors (35). The infiltration of tumor cells, the immune functions, as well as the expression of immune checkpoint inhibitors could influence the prognosis of cancer patients as well as predict the response to immunotherapies (35, 36). Manipulating the expression of m6A regulators or lncRNAs could modify the immune microenvironment and immune-related biological processes (15, 29). Here we found that there were significant differences in the immune cell infiltrations, immune functions, and expression of immune checkpoint inhibitors between the high- and low-risk groups. Thus, further understanding of the m6A-regulated lncRNAs and immune microenvironment could improve the immunotherapy strategies for LUSC patients. Jin et al. construct an m6A-related signature which could predict the response to immunotherapy in adrenocortical carcinoma (37). Wang et al. constructed an m6A-related lncRNA signature which could discriminate patients with response to immune checkpoint inhibitor in gastric cancer (28).

Moreover, given that multivariate Cox regression analysis showed that AL122125.1 demonstrated a better prognostic value than HORMAD2-AS1 and could independently predict the survival of LUSC patients, we thus further explored the expression and biological function of AL122125.1 in LUSC. The upregulated expression of AL122125.1 was correlated with poor overall survival of LUSC patients. The GO and KEGG enrichment analyses revealed that the biological functions of AL122125.1 were tumor-related. There are some limitations of this study. Firstly, the AUC values of the risk model and other clinical risk factors (age, gender, and stage) were below 0.6. Among them, the AUC value of the risk model was the highest. These might be due to the high heterogeneity of LUSC. Thus, the validation of expression and biological functions of AL122125.1 still needs to be carried out in our clinical samples—cellular and mice experiments. Secondly, AL122125.1 and the corresponding m6A regulators need to be further explored by using cellular experiments.

In conclusion, we identified an m6A-related lncRNA prognostic risk model from the TCGA-LUSC cohort. This risk model demonstrated close associations with the immune microenvironment, which may provide novel insights into LUSC therapeutic strategies and guide effective immunotherapy.

## Data Availability Statement

The datasets presented in this study can be found in online repositories. The names of the repository/repositories and accession number(s) can be found in the article/supplementary material.

## Author Contributions

CW and LL conceived the project. GL supervised this project. CW and LW conducted the data analysis. LL and CW drafted the manuscript. LW and MG revised the manuscript. All authors contributed to the article and approved the submitted version.

## Funding

This research was funded by the Natural Science Foundation of Guangdong Province, China (no. 2021A1515011113), the Guangzhou Science and Technology Program (no. 201904010427), and the Guangzhou General Science and Technology Project of Health and Family Planning (grant number 20201A011007).

## Conflict of Interest

The authors declare that the research was conducted in the absence of any commercial or financial relationships that could be construed as a potential conflict of interest.

## Publisher’s Note

All claims expressed in this article are solely those of the authors and do not necessarily represent those of their affiliated organizations, or those of the publisher, the editors and the reviewers. Any product that may be evaluated in this article, or claim that may be made by its manufacturer, is not guaranteed or endorsed by the publisher.

## Abbreviations

M6A, N6-methyladenosine; lncRNA, long noncoding RNA; LUSC, lung squamous cell carcinoma; TCGA, The Cancer Genome Atlas; ROC, receiver operating characteristic; AUC, area under the curve; GO, Gene Ontology; KEGG, Kyoto Encyclopedia of Genes and Genomes; NSCLC, non-small cell lung cancer.
